# Influence of Surface Roughness Spatial Variability and Temporal Dynamics on the Retrieval of Soil Moisture from SAR Observations

**DOI:** 10.3390/s90100463

**Published:** 2009-01-13

**Authors:** Jesús Álvarez-Mozos, Niko E.C. Verhoest, Arantzazu Larrañaga, Javier Casalí, María González-Audícana

**Affiliations:** 1 Department of Projects and Rural Engineering, Public University of Navarre, Los Tejos, Arrosadia s/n, 31006 Pamplona, Spain; E-Mails: jcs@unavarra.es; maria.audicana@unavarra.es; 2 Laboratory of Hydrology and Water Management, Ghent University, Coupure links 653, B-9000 Ghent, Belgium; E-Mail: Niko.Verhoest@UGent.be; 3 Department of Territorial Information Systems, TRACASA, Cabárceno 6, 31621, Sarriguren, Spain; E-Mail: alarranaga@tracasa.es

**Keywords:** Synthetic Aperture Radar, Soil moisture retrieval, Surface roughness

## Abstract

Radar-based surface soil moisture retrieval has been subject of intense research during the last decades. However, several difficulties hamper the operational estimation of soil moisture based on currently available spaceborne sensors. The main difficulty experienced so far results from the strong influence of other surface characteristics, mainly roughness, on the backscattering coefficient, which hinders the soil moisture inversion. This is especially true for single configuration observations where the solution to the surface backscattering problem is ill-posed. Over agricultural areas cultivated with winter cereal crops, roughness can be assumed to remain constant along the growing cycle allowing the use of simplified approaches that facilitate the estimation of the moisture content of soils. However, the field scale spatial variability and temporal variations of roughness can introduce errors in the estimation of soil moisture that are difficult to evaluate. The objective of this study is to assess the impact of roughness spatial variability and roughness temporal variations on the retrieval of soil moisture from radar observations. A series of laser profilometer measurements were performed over several fields in an experimental watershed from September 2004 to March 2005. The influence of the observed roughness variability and its temporal variations on the retrieval of soil moisture is studied using simulations performed with the Integral Equation Model, considering different sensor configurations. Results show that both field scale roughness spatial variability and its temporal variations are aspects that need to be taken into account, since they can introduce large errors on the retrieved soil moisture values.

## 1. Introduction

Surface soil moisture (*SM*) is a variable that plays a crucial role in many processes occurring at the soil-atmosphere interface. The knowledge of the moisture content of the soil over a field or a catchment can be very helpful for hydrological, agronomical and meteorological applications [1-2]. Being such an extremely dynamic variable, the possibility of achieving its estimation by means of remote sensing observations is very interesting for many applications [2]. At present, active microwave (radar) sensors represent the best alternative for a remote *SM* estimation for hydrologic and agronomical applications [1]. The backscattering coefficient (*σ*^0^), obtained from radar sensors, is directly related to the dielectric properties of the soil surface being observed, which is mainly dependent on its moisture content [3]. Furthermore, the spatial resolution of SAR sensors [4] and their ability to observe the surface through clouds are aspects that make them interesting for hydrological and agronomical applications.

Radar-based *SM* retrieval has been intensively studied in the last decades. Different approaches have been developed and used with varied success [1]. Among others, the most rigorous approach seems to be the use of electromagnetic scattering models that simulate the surface backscattering process [3, 5-11]. Those models can be inverted to retrieve *SM*. Several models have been proposed for bare soils or sparsely vegetated surfaces. At present, the Integral Equation Model (IEM) [5] and the Geometrical Optics Model (GOM) [3], both physically-based models, are the most frequently used algorithms for soil moisture retrieval [12-14]. The former is applicable on smooth or medium roughness conditions and the latter on rough or very rough surfaces. Consequently, both models cover the range of roughness conditions that can be expected over most agricultural surfaces. In addition, these theoretical models have been validated against observations acquired on experimental plots and laboratory settings verifying the adequacy of their predictions as long as their applicability conditions are met [5, 15, 16].

However, the application of the IEM to natural conditions has been so far problematic [7, 12, 13, 17-22]. Frequently, the poor results obtained have been related to the influence of soil roughness on the backscatter [23]. Furthermore, it has been reported that an accurate field measurement of the required roughness parameters (standard deviation of surface heights *s* and correlation length *l*), in particular *l*, is extremely difficult to perform [23-27].

In some circumstances the influence of surface roughness can be simplified. For instance, over agricultural areas where winter cereals are grown, the soil surface remains untilled from sowing (end of October) till harvest (end of June). During this period, surface roughness can be assumed constant and *SM* inversion can be simplified assuming that *σ*^0^ variations are only a consequence of *SM* dynamics. Of course, over those agricultural areas the vegetation cover can influence the SAR response and complicate the *SM* retrieval [28, 29]. However, in the first stages cereal crops develop slowly and it takes some months until their influence on the SAR signal is significant [30]. Therefore, during the agricultural inactive period (approximately from November till March for the Spanish watershed studied) the assumption of constant roughness conditions and the use of simplified *SM* inversion approaches seems a good choice. In addition, most of the precipitation over temperate climate regions occurs during those autumn and winter months, making the estimation of soil moisture and its dynamics during this period very interesting for hydrological applications.

In autumn and winter periods, severe storms can cause a smoothening of the soil surface, resulting in variations in the surface roughness conditions. The study of the soil surface smoothening caused by precipitation is not new. Many investigations have been carried out on this subject, mostly by soil erosion scientists willing to characterize the detachment and transport of soil particles by rainfall and runoff [31]. Indeed, surface roughness is an important variable on hydrological and erosion processes and its knowledge is important for many simulation models in these fields. Most of the studies performed to evaluate the soil surface smoothening by precipitation were conducted on laboratory conditions or experimental plots using artificial rainfall simulators. Results evidenced a reduction on the surface roughness as a result of the disintegration and relocation of soil aggregates caused by precipitation. The reduction of the standard deviation of surface heights has been described as an exponential function of the accumulated precipitation or the accumulated kinetic energy of precipitation [31]. This type of exponential decay functions have been incorporated to widely known erosion models such as the EUROpean Soil Erosion Model EUROSEM [32].

The above mentioned studies were performed using rainfall simulators that generated very high precipitation rates which are far more intense than observed during normal precipitations of temperate areas [31]. So, it is interesting to evaluate the variation of roughness under real precipitation conditions. Besides, even if the reduction of the parameter *s* has been frequently evidenced, the evolution of the correlation length *l* has barely been studied (e.g. [24]), and little is known on its variations with precipitation. Consequently, it is important to evaluate the variations of surface roughness that can occur over those winter cereal growing areas and to assess their influence on the estimation of soil moisture from radar data.

Roughness parameters are also very variable in space [31, 33-35]. Generally, SAR-based *SM* estimations are made at the field or catchment scale. Point or pixel estimates are not realistic due to the influence of speckle and roughness spatial variability, therefore aggregation to the field or catchment level is preferable. Nevertheless, catchment and field scale *SM* estimates are still very valuable for most applications. Yet, roughness spatial variability can still exert a strong influence on the retrieval of *SM* at those scales. The measurement or estimation of field average roughness parameters can be extremely difficult over agricultural and natural surfaces, and the influence of an inaccurate estimation on *s* and *l* can have consequences on the retrieval of *SM*. Yet the quantification of this impact has never been explicitly evaluated to our knowledge.

In this study, we present the results of an intensive campaign of roughness ground measurements and analyze the field scale variability and temporal dynamics of roughness parameters during a winter cereal season. Next, their implications on the estimation of *σ*^0^ using the IEM and on the subsequent retrieval of *SM* from radar data are evaluated by means of a synthetic analysis. In the analysis, different sensor configurations (polarization, frequency and incidence angle) and soil moisture conditions are considered to investigate any possible relation between the conditions of the observations and the sensitivity to roughness variations.

## 2. Materials and Methods

### 2.1. Test site

The research was carried out over a small agricultural watershed located in the Spanish region of Navarre called *La Tejería*. This watershed is part of the Experimental Agricultural Watershed Network of Navarre, created by the local Government of Navarre in 1993 and aimed at studying the impact of agriculture on the hydrological resources [36].

The geographical coordinates of the watershed outlet are 42°44′10.6″N and 1°56′57.2″W. The watershed covers approximately 170 ha with homogeneous slopes of about 12%, and an altitude ranging from 496 to 649 m. Its climate is humid submediterranean, with a mean annual temperature of 13°C. The average annual rainfall is about 700 mm, distributed over approximately 105 days.

The most common soils are *Tipic Xerochrepts*, which are less than 1 m deep. Those soils have Silty-Clay texture (43% clay, 5% sand, 52% silt) and cover most of the hillslopes. The watershed is almost completely cultivated and the hedgerows and streams are the only areas covered by natural vegetation. The main crops are winter cereals (wheat, barley and oat) and less frequently rain fed vegetables (chickpeas and beans) and sunflower. Normally, the growing cycle starts in September when soil preparation and tillage operations are performed. Soil preparation operations consist of a deep tillage operation (usually moldboard plowing); a second operation to break the soil clods and refine the surface using a spike harrow (this harrowing operation is usually applied twice); finally, the cereal is sown (approximately around October) and, in some few fields, the soil surface is compacted afterwards using a roller.

### 2.2. Ground measurements

Surface roughness measurements were performed during the agricultural year 2004-2005. Ten control fields were selected over the catchment, with field sizes ranging from 3.0 ha to 7.3 ha. Ground measurements were performed on eight dates: 22/09/2004, 08/10/2004, 24/10/2004, 12/11/2004, 28/11/2004, 17/12/2004 and 01/03/2005. Four profiles were acquired per field on each date (with few exceptions, see Table 1), making a total of 264 profiles. On the first three dates different tillage classes were measured, from the fourth date onward, the cereal crop was sown and the tillage state was classified as ‘Seedbed’ (Table 1). After March, the vegetation cover is, in general, sufficiently developed to protect the soil surface from the impact of precipitation, so no further measurements were acquired later in the season. In classes where clear tillage rows were observed, profiles were acquired in parallel to the rows, in order to reflect the random component of roughness. Measurements perpendicular to the rows were not performed, since the periodic pattern introduced by the rows should be removed from the obtained roughness profile, and accounted for separately in the backscatter model [3].

**Figure 1.**
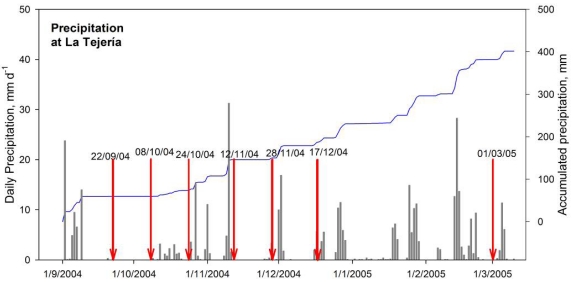
Daily and accumulated precipitation recorded during the studied period. Measurement dates are indicated by red arrows. Dates are given in dd/mm/yyyy format.

During the research period, the accumulated precipitation was 382 mm, which can be considered normal in the region [36]. Precipitations were scarce on the first dates and more frequent during winter months (Figure 1).

**Figure 2.**
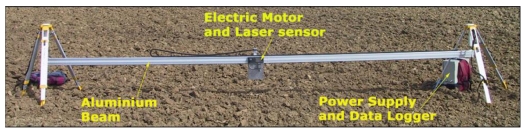
Main components of the laser profilometer.

Surface roughness measurements were performed using a non-contact profilometer that incorporates a laser sensor to measure the distance from a reference beam to the soil surface (Figure 2). The main advantages of the instrument comparing to other roughness measuring methods consist of the facts that the soil surface remains unchanged after the measurement, the profile data are directly downloaded, omitting the need of post-processing, and the very high accuracy of the instrument.

The laser profilometer consists of an aluminium beam, attached to two tripods at both ends (Figure 2). A laser sensor is placed on a small carriage that is moved along the beam driven by a small electric motor. The laser sensor has a vertical accuracy of 1 mm and is programmed to acquire and store height data every 5 mm. The total length of acquired profiles is 5 m, and the beam can be dismantled in two pieces to be more easily handled and transported. Two plastic racks are attached to the aluminium beam; the former is used by the motor gear to move the carriage and the latter to provide a distance reference to the sensor from which the instrument infers when measurements need to be stored. The instrument is connected to a power supply unit that also contains the data logger.

The processing of the profiles acquired is simple and fast. Once profiles are downloaded from the data logger to a PC, the beam deformation is corrected using a parabolic curve fitted to a set of reference measurements acquired in the lab. Afterwards, any shape or trend corresponding to the surface topography is removed. Finally, roughness parameters (*s* and *l*) are calculated. Field average *s* values were obtained as the arithmetic mean of individual *s* data, whereas average *l* values were derived from the average autocorrelation function (*ACF*) calculated using the *ACF*s of individual profiles.

### 2.3. Backscatter model

The IEM was used in order to evaluate the influence of roughness variations in the backscattering coefficient of surfaces. In the present research, a simplified version of the IEM was applied which considered only the single scattering term of the backscattered wave [5, 17]. This version is applicable to surfaces with small to moderate roughness conditions or at low to medium frequencies, having a validity range restricted by *ks* < 3 and *m* < 0.4; with *k* being the wave number and *m* the surface roughness slope, which for exponentially autocorrelated surfaces equals *s/l*. The description of the model can be found in the literature (for instance in [5, 13, 17]).

It should be remarked that the aim of the study is not to compare the accuracy of IEM estimations with observed SAR data. The intent is to evaluate the importance of roughness variations. Therefore, no actual SAR data were analysed in this study. A basic assumption of the study is that the IEM provides adequate backscatter simulations as long as its applicability conditions are met.

The IEM calculates the backscattering coefficient from a surface given its roughness parameters (*s, l*) and exponential *ACF* (valid for different soil tillages [24]), its dielectric constant (*ε*) and the scene acquisition parameters: frequency, incidence angle and polarization. In the present research, *ε*was calculated through the Dielectric Mixing Model [37] using *SM*, soil texture and temperature data.

After inverting the model, the dielectric constant, and hence *SM*, can be retrieved from *σ*^0^ observations given the roughness parameters. In this paper the inversion was performed using a look-up-table type scheme. In order to prevent the model from predicting physically impossible *SM* values, the inverted *SM* values were limited to a range between 0.001 cm^3^cm^-3^ and 0.600 cm^3^cm^-3^.

### 2.4. Synthetic analysis

Synthetic analyses based on backscatter models have been used frequently to circumvent the rare availability of extensive SAR observations coincident with high amounts of accurate ground measurements. In the SAR-based soil moisture retrieval literature synthetic studies based on the IEM have been performed with several objectives: (1) to derive or to validate simplified (semi-empirical) models [6, 7, 9, 10, 38]; (2) to develop statistical retrieval methods based on neural networks [12, 39-41]; Bayesian techniques [28, 42]; or possibilistic algorithms [43] and fuzzy rule-based models [44]; (3) to perform sensitivity and error analyses [10, 13, 17, 20, 23, 45-48]; and (4) to analyze the influence of roughness measurements' profile length on the calculated backscattering coefficient [49-50].

Ideally, synthetic studies should be completed with experimental observations, so the use and interpretation of their results must be cautious. Nonetheless, they are useful to reveal trends or to test different hypothesis, especially in cases where different parameters interact or vary and the interpretation of experimental SAR data becomes difficult.

The synthetic analysis, discussed in this paper, was focused on seedbed fields where the simplifying approaches mentioned in the introduction (constant roughness conditions) could be applied. Several sensor configurations were considered in order to assess their influence on the accuracy of the retrievals. Selected configurations corresponded to those of available spaceborne SAR sensors (i.e. ERS-2, RADARSAT-1/-2, ENVISAT/ASAR and ALOS/PALSAR), thus the results obtained can be linked to different sensors. Regarding polarization, HH and VV configurations were considered since they are more adequate for soil moisture retrieval than cross-polarized configurations [3]. Three incidence angle conditions were used: 15°, 30° and 45°, which correspond to steep, medium and large incidence angles selectable in most sensors. Finally, two frequencies were selected, C-band (5.3 GHz) and L-band (1.27 GHz).

Three different soil moisture conditions were tested: 0.05 cm^3^cm^-3^ (dry), 0.20 cm^3^cm^-3^ (medium) and 0.35 cm^3^cm^-3^ (wet), in an attempt to evaluate whether the influence of roughness was related to the moisture of soils being observed. Finally, the following soil characteristics (necessary to transform *SM* values to dielectric constant) were considered: sand fraction = 10 %, clay fraction = 35 %, bulk density = 1.4 g cm^-3^ and soil temperature = 10 °C.

First, IEM simulations were carried out to evaluate the influence of the field scale variability of the roughness parameters on *σ*^0^ and on the retrieved *SM*. Therefore, field average *σ*^0^ values were calculated using average roughness parameters which were then compared to the *σ*^0^ values calculated from *s* and *l* of the individual profiles. The differences were quantified calculating the root mean square error (*rmse*) in dB. In the inverse simulations, *SM* was retrieved using field average *s* and *l* values and *σ*^0^ obtained from individual profile data. The deviations from the initially set *SM* values (0.05, 0.20 or 0.35) were calculated, those can be an indicator of the influence of field scale roughness variability on the retrieved *SM* values.

Next, the IEM was used to convert the temporal variations of roughness to variations on the *σ*^0^ and on the retrieved *SM*. In this case, for each field the *σ*^0^ calculated for the first seedbed date was compared to those calculated on the subsequent dates. The variations on *σ*^0^ calculated this way, were only caused by the temporal variations of roughness since all the other parameters (*SM*, sensor configuration, etc.) were kept constant. In the inverse mode, *SM* was retrieved using *s* and *l* measurements of the first seedbed date and *σ*^0^ values obtained using field average *s* and *l* values measured on the subsequent dates. The differences between the retrieved *SM* values and the ones initially set are a consequence of the temporal variations of roughness.

## 3. Results

### 3.1. Influence of the spatial variability of roughness

#### 3.1.1. Roughness measurements

**Figure 3.**
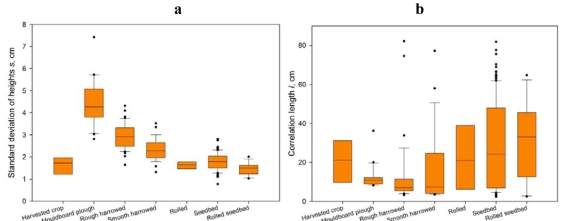
Boxplots of the ground measured roughness parameters. a) Standard deviation of surface heights *s* and b) Correlation length *l*.

Roughness parameters behaved differently depending on the tillage class. Figure 3 represents the boxplots of ground measured *s* and *l* values.

It can be observed that *s* took larger values as the tillage class became rougher. Generally, fields of different classes had different *s* values, although the variability of *s* was large in some classes (particularly the rough ones). The behaviour of *l* was different, demonstrating for most of the classes a very large variability and a similar variation range. For this study, rough classes had a lower variability range than smooth classes.

In Table 2, field scale average values are presented. Focusing on the field scale spatial variability of the parameters, it can be observed that the average coefficients of variation ranged from 16% to 25% for parameter *s* and from 38% to 94% for parameter *l*.

**Figure 4.**
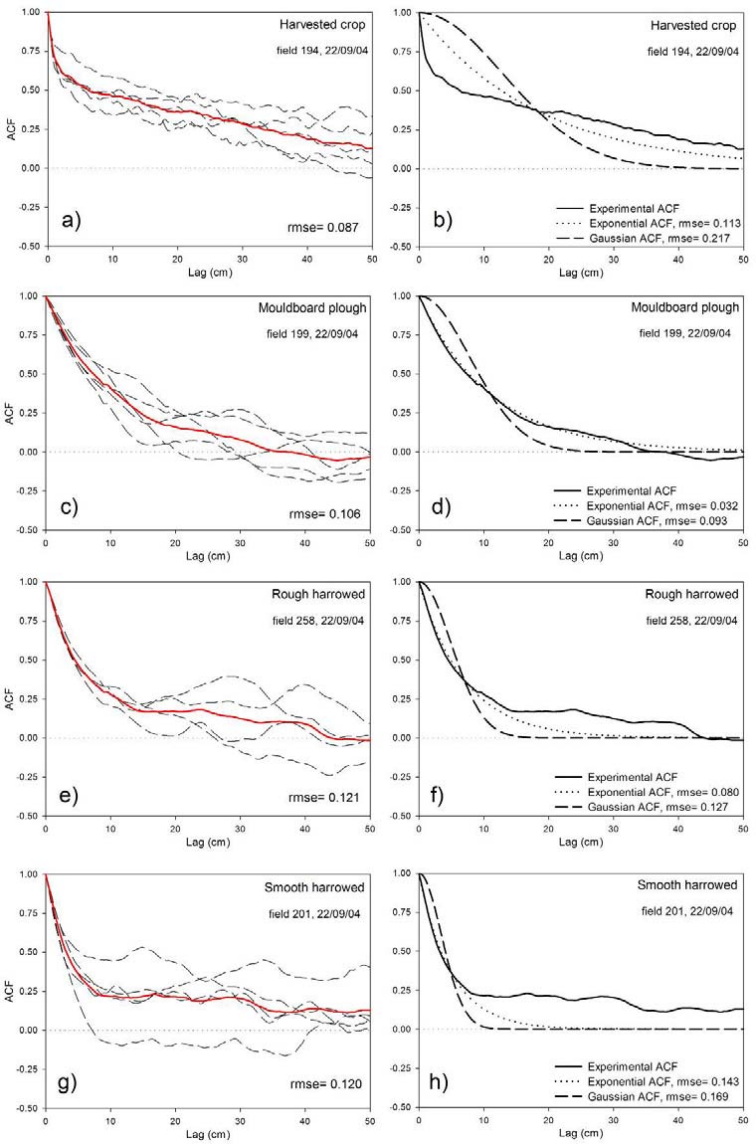

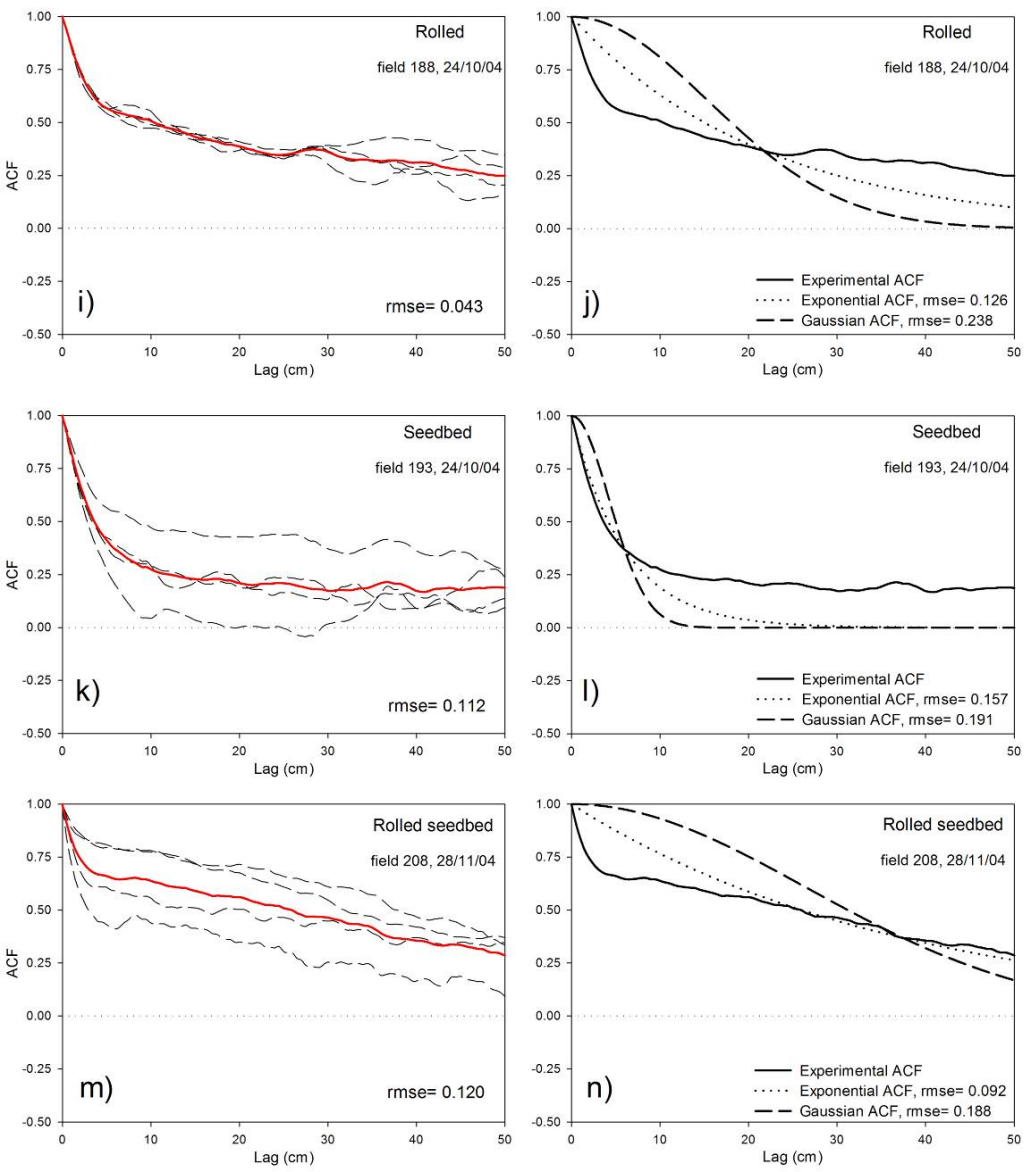
Autocorrelation functions (*ACF*) calculated for fields of different tillage classes. For each field the individual and average *ACF* are plotted (in red) and the root mean square error (*rmse*) of *ACF* is given. The obtained *ACF)* are fitted to exponential and Gaussian functions and the *rmse* of their fit is also indicated.

Figure 4 depicts the autocorrelation functions (*ACF*) obtained for fields belonging to the different tillage classes studied. For each field the mean *ACF* was computed averaging the individual *ACF*s. Next, the root mean square error (*rmse*) of the field average *ACF* was calculated. This *rmse* value illustrates the spatial variability of the *ACF*s. The field average *ACF* was fitted to the exponential and Gaussian functions and the *rmse* of their fit was computed. The plots show that the variability of the *ACF*s can be large, especially at higher lags. To avoid the impact of the larger variability at higher lags the calculation of the *rmse* values was restricted to a lag up to 50 cm.

The plots illustrated in Figure 4 show that the spatial variability of *ACF*s can be high in both smooth and rough surfaces. In smooth surfaces, the *ACF* tends to fall quickly at small lags due to high frequency roughness components. But the slope of the function decreases soon leading in some cases to very large *l* values. For rough surfaces, the decay is generally more homogeneous with a gradual reduction of the slope. This type of *ACF* represents surfaces that are rough in both high and low frequency domains. In all cases, the field average *ACF* fits better the exponential function than the Gaussian. The fit to the exponential function is particularly good for rough surfaces. For smooth surfaces, exponential and Gaussian functions do not provide an adequate description of the initial decay and the sudden reduction of the slope of the *ACF*s.

For each tillage class the average of the field *ACF̅ rmse* was calculated and the values obtained are represented in Table 3. It can be observed that the variability of the *ACF*s was similar in the different classes, with values ranging from 0.085 (‘Rolled seedbed’) to 0.130 (‘Seedbed’). In fact, rough and smooth classes yielded similar *rmse* values, indicating a substantial variability of the *ACF*s in all cases. Table 3 also gathers the *rmse* values of the *ACF* fit to the exponential and Gaussian functions. It can be observed that the fit to the exponential function is better (lower *rmse*) in all cases. Generally, rough classes fit better the exponential function than the smooth ones, except for the class ‘Rolled seedbed’.

The discrepancies between the experimental and the exponential *ACF*s illustrated here show that assuming an exponential *ACF* in the retrieval of *SM* could be a significant source of errors. However, the quantification of these errors in terms of the modelled *σ*^0^ or the retrieved *SM* has never been done before, as far as the authors know. In any case, this quantification is out of the scope of this paper, so the results described in the next section assume an exponential *ACF* and do not represent the influence of the discrepancies between the experimental *ACF* and the exponential function.

In summary, the average standard deviation of *s* and *l* on seedbed fields was 0.33 cm and 19.24 cm respectively. In the following sections, the influence that this variability exerts on the simulated backscattering coefficient values and on the retrieved *SM* estimates, is assessed using IEM simulations.

**Figure 5.**
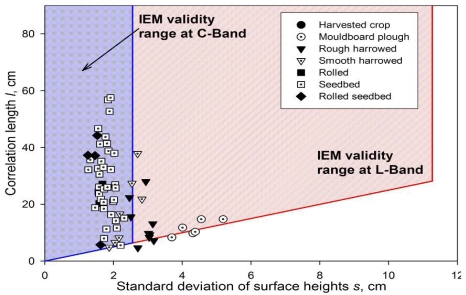
IEM validity range at both C- and L-bands and field average roughness parameters measured for the different tillage classes.

Figure 5 represents the field average *s* and *l* values measured and the IEM validity range for both C- and L-bands. It can be observed that most seedbed fields are within the C-band range, whereas most of the fields of rougher classes are not. At L-band the validity range is much wider and covers most of the measured fields. However, for some fields the *s/l <* 0.4 condition does not hold, causing their exclusion from further analysis.

#### 3.1.2. Influence on *σ*^0^ estimations

To evaluate the influence of the field scale roughness variability on *σ*^0^, the IEM was run using field average roughness parameters first and then the parameters measured on each profile individually were applied. This resulted in field average and individual backscatter values, called *σ̄*^0^ and *σ*^0^_i_ respectively.

**Figure 6.**
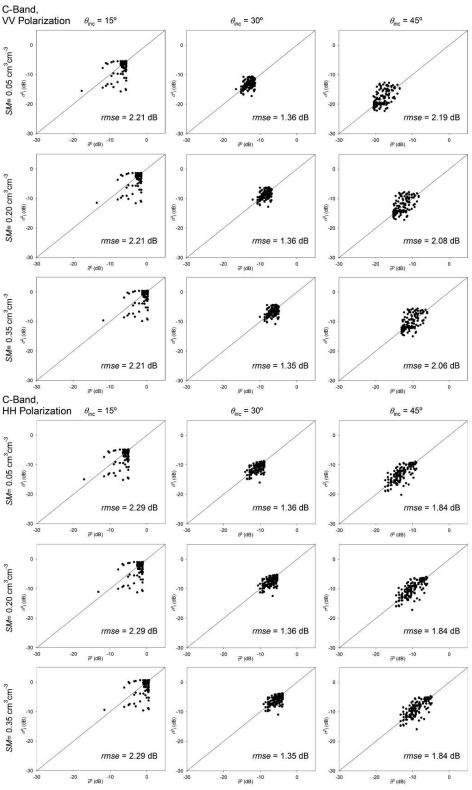
Comparison between field average (*σ̄*^0^) and individual (*σ*^0^_i_) backscattering coefficient values calculated with the IEM considering C-band frequency and different *θ*_inc_ and *SM* conditions. The differences are due to the intra-field variability of roughness parameters, the root mean square error (*rmse*) is given.

**Figure 7.**
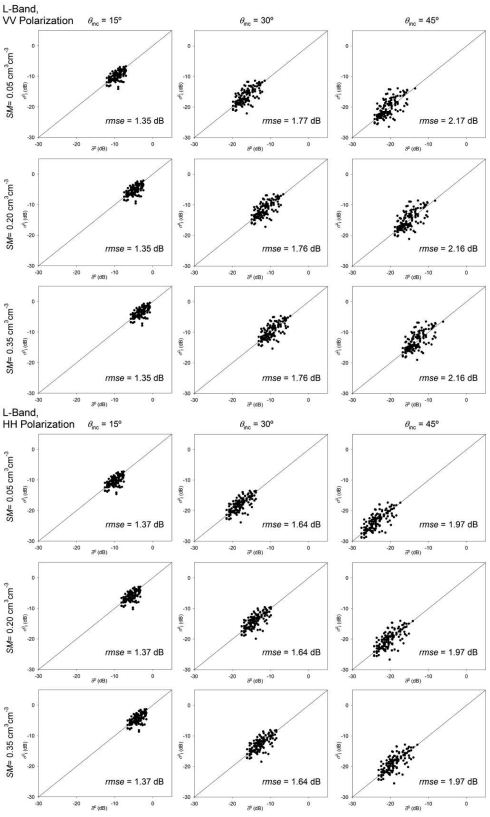
Comparison between field average (*σ̄*^0^) and individual (*σ*^0^_i_) backscattering coefficient values calculated with the IEM considering L-band frequency and different *θ*_inc_ and *SM* conditions. The differences are due to the intra-field variability of roughness parameters, the root mean square error (*rmse*) is given.

The comparison between *σ*^0^_i_ and *σ̄*^0^ (Figures 6 and 7) illustrates the influence of roughness spatial variability on *σ*^0^. It should be remarked that the considered sensor configuration and soil characteristics were exactly the same for every field. This means that *σ̄*^0^ (abscissa in Figures 6 and 7) varied only due to the differences in the field average *s* and *l* values from field to field. This field average *σ̄*^0^ showed a variation range between 5 and 10 dB. This result indicates that assuming tillage class average or reference roughness values could imply high inaccuracies in the modelling of *σ*^0^.

The intra-field spatial variability of roughness caused *σ*^0^_i_ values to be very different from their corresponding field average *σ*^0^ values in some cases. The root mean square error (*rmse*) values obtained ranged from 1.36 to 2.29 dB. Highest errors were observed at 45° incidence angle in both C- and L-bands and at 15° in C-band.

The same *rmse* values were obtained for different *SM* conditions, so the influence of roughness spatial variability seems not to be affected by the moisture conditions of soils. Very similar errors were observed also for both VV and HH polarizations. The *rmse* was slightly higher on VV for 45° incidence angles but this difference did not reach 0.5 dB. In the other cases, both polarizations yielded very similar *rmse* values.

The incidence angle showed a significant influence on the obtained *rmse* values. For C-band simulations (Figure 6), *rmse* was minimum at 30° and increased at both 15° and 45°. In the case of L-band (Figure 7), *rmse* increased with the incidence angle, and highest errors were observed at 45°.

**Figure 8.**
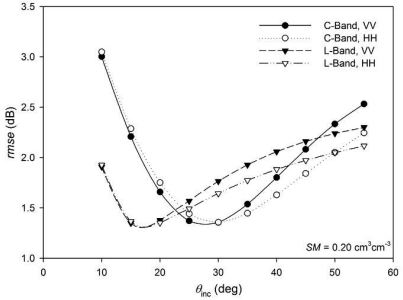
Root mean square errors (rmse) obtained between individual (*σ*^0^_i_) and field average (*σ̄*^0^) backscatter values considering different incidence angles.

In order to analyse the influence of the incidence angle in more detail, an additional simulation was conducted varying *θ*_inc_ from 10° to 55° with a 5° step and considering average moisture conditions (*SM=*0.20 cm^3^cm^-3^) for HH and VV polarization and both C- and L-bands. Figure 8 represents the *rmse* values obtained as a function of *θ*_inc_ for the different configurations. The trend was similar in all cases, at low *θ*_inc_ values errors were high, as the *θ*_inc_ increased *rmse* first decreased to a minimum and then increased.

Minimum *rmse* values were observed at 15°-20° in L-band and 25°-30° in C-band. At low *θ*_inc_ values errors were significantly higher in C-band, but at medium *θ*_inc_ the opposite was evidenced, this is in agreement with previous studies [19], which evidenced higher roughness sensitivity at L-band than at C-band on SIR-C/X SAR observations with 26.4° incidence angle. In our study, large *θ*_inc_ values gave *rmse* values higher than 2.0 dB in all cases, which were slightly higher in VV polarization than in HH.

#### 3.1.3. Influence on the retrieved *SM*

A similar analysis was performed running the inverse IEM algorithm. This time, field average *σ̄*^0^ values were used as input along with the roughness parameters of the individual profiles. The inverted *SM* values should be equal to the initially assumed conditions (0.05, 0.20 or 0.35 cm^3^cm^-3^), so the discrepancies observed (evaluated as *rmse* values) were considered a consequence of the field scale roughness variability.

**Figure 9.**
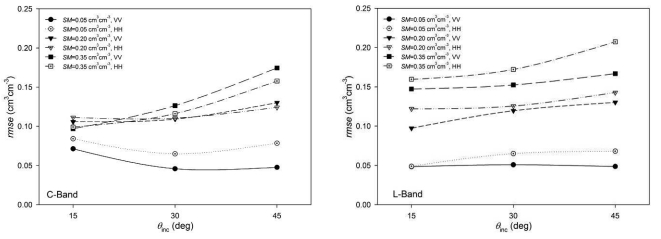
Root mean square errors (*rmse*) obtained in the *SM* inversion as a function of the incidence angle *θ*_inc_.

Figure 9 shows the *rmse* values obtained as a function of *θ*_inc_ for both bands and different *SM* conditions. It can be observed that independent from the incidence angle trend (which is similar to the one observed in the forward simulations), *rmse* values were different depending on the *SM* conditions. For wet soils, *rmse* values were higher, especially on L-band and on C-band with large *θ*_inc_. In fact, for wet conditions, retrieved *SM* values were sometimes even higher than the fixed upper limit (0.60 cm^3^cm^-3^).

**Figure 10.**
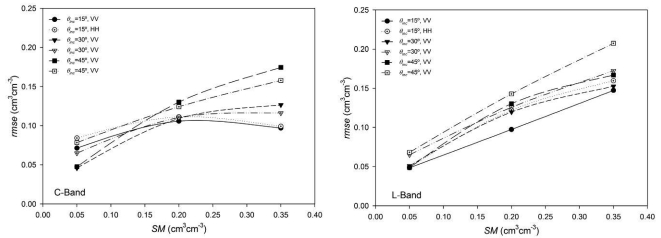
Root mean square errors (*rmse*) obtained in the *SM* inversion as a function of the considered *SM* conditions.

If we look more closely to the *rmse* as a function of *SM* (Figure 10) we can observe a clear relationship, especially in L-band. Errors were close to 0.05 cm^3^cm^-3^ in the driest case and they increased with moisture, sometimes reaching values around 0.15 cm^3^cm^-3^. This phenomenon could be a result of the reduced sensitivity of *σ*^0^ to soil moisture for higher *SM*-values. A given error in *σ*^0^ produces a certain error on the retrieved *SM* on dry conditions but this error could increase substantially over wet conditions. Similar results have been reported in the literature [13, 17, 33]. This behaviour seems to be more pronounced at L-band, resulting in higher *SM* retrieval errors on wet conditions than at C-band.

In summary, the results of this synthetic analysis indicate that the intra-field spatial variability of roughness could severely affect the inversion of *SM* from SAR data. Unless roughness parameters are very accurately measured, retrieval errors could easily reach 0.10 cm^3^cm^-3^, and even more on wet conditions. Consequently, the measurement or characterization of the roughness parameters for each field needs to be carried out with high detail in order to retrieve useful *SM* estimates.

### 3.2. Influence of the temporal dynamics of roughness

#### 3.2.1. Roughness measurements

**Figure 11.**
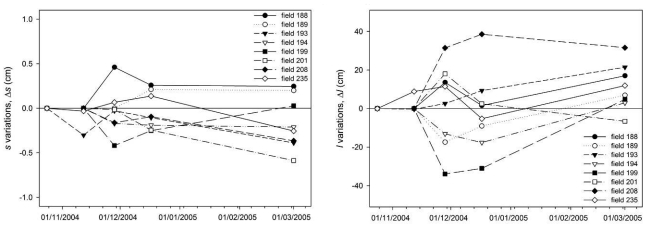
Temporal variations of roughness parameters for seedbed fields.

The temporal variations of *s* and *l* were only evaluated for seedbed fields. Measurements corresponding to five dates were analysed (from 24/10/2004 to 01/03/2005). In Figure 11, the temporal variations of both roughness parameters are illustrated. Variations were computed for each field in reference to the measurements of the first seedbed date, so negative values represent a reduction on the parameter and positive values an increment.

**Figure 12.**
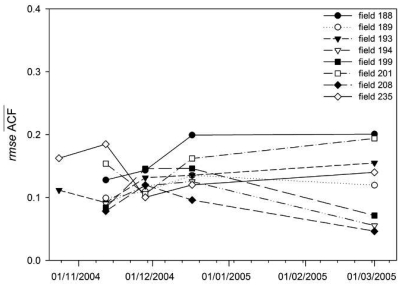
Temporal variations of ACF variability for seedbed fields, measured in terms of the root mean square error (*rmse*) of the field average autocorrelation function (*ACF*). Numbers 188 to 235 label the different fields measured.

The temporal evolution of the *ACF* variability (*rmse* of the field *ACF*) was also analyzed. Figure 12 depicts the *rmse* values calculated for each seedbed field *ACF* on the different dates. It can be observed that the variability of the *ACF*s was similar in the different dates. For some fields the *rmse* value decreased whereas for others it increased. Therefore, even if surfaces roughness appeared to smoothen with time the variability of the *ACF*s did not change significantly.

**Figure 13.**
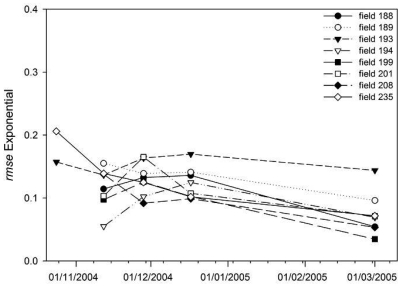
Temporal variations of the goodness of fit to the exponential *ACF* for seedbed fields, measured in terms of the root mean square error (*rmse*) between the field average *ACF* (*ACF*) and the exponential function. Numbers 188 to 235 label the different fields measured.

On the other hand, the fit to the exponential function improved with time. Figure 13 plots the *rmse* of the fit to the exponential function for the different fields as a function of time. It can be observed that the *rmse* values obtained decreased in most of the fields. This means that the smoothening caused by precipitation resulted in more exponential-like surfaces, probably due to the reduction of the high frequency roughness components of the surface that caused the steep fall at the beginning of the *ACF*s illustrated in Figure 4 for smooth classes.

#### 3.2.2. Influence on *σ*^0^ estimations

The recorded temporal variations of *s* and *l* could cause a certain variation on *σ*^0^. To assess this variation, the backscatter values calculated considering constant, or initial, roughness parameters (*σ*^0^_0_), were compared to those calculated using *s* and *l* values of each date (*σ*^0^_i_). The differences were computed as Δ*σ*^0^=*σ*^0^_0_ - *σ*^0^_i_. The objective was thus to assess whether the roughness dynamics caused significant variations on *σ*^0^.

**Figure 14.**
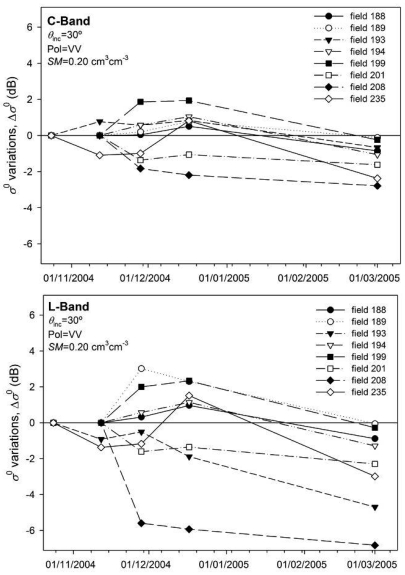
Backscattering coefficient variations (Δ*σ*^0^) in C- and L-bands caused by the temporal variations of roughness parameters on seedbed fields.

Figure 14 shows the Δ*σ*^0^ values computed for both bands at 30° incidence angle, VV polarization and 0.20 cm^3^cm^-3^ as a function of time. Variations were similar to those of the roughness parameters themselves; on the first dates *σ*^0^ increments and reductions were observed with no clear trend. But the roughness variations on the last date caused a clear reduction in *σ*^0^, especially in L-band. In these conditions both *s* reductions and *l* increments made *σ*^0^ decrease, so even if roughness temporal variations seemed minor, their impact on *σ*^0^ could be important.

The backscatter variations were calculated for all different configurations considered but they are not plotted for the sake of conciseness. Instead, the total variations between the first and last dates were calculated for the different fields and their average, minimum and maximum values for the different conditions are represented in table 4. As the same Δ*σ*^0^ values were obtained at different *SM* conditions (as in section 3.1.2), the numbers in table 4 are not restricted to any *SM* content.

**Figure 15.**
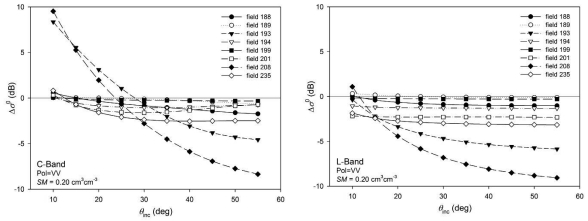
Backscattering coefficient variations (Δ*σ*^0^) for increasing incidence angles and different test fields.

It can be observed that reductions were more significant at large incidence angles, whereas at 15° increments were observed especially for C-band. This could be a consequence of the smoothening of the surface, that caused a specular-like type scattering behaviour producing higher *σ*^0^ values for steep incidence angles and lower *σ*^0^ values for large angles. This effect is further illustrated in figure 15 and it is particularly evident at C-band in fields with strong *s* reductions and *l* increments (fields 193 and 208). The backscatter variations are minimum at 25°-30° in C-band and 15°-20° in L-band.

#### 3.2.3. Influence on the retrieved *SM*

**Figure 16.**
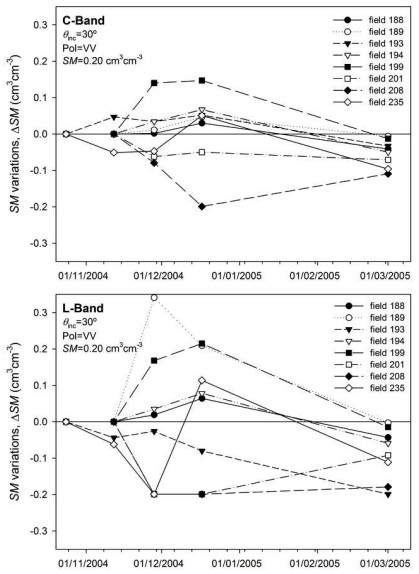
Inverted *SM* variations (Δ*SM*) in C- and L-bands caused by the temporal variations of roughness parameters on seedbed fields.

In order to evaluate the influence of temporal variations of roughness on the inverted *SM*, the IEM was run assuming constant roughness parameters (*s* and *l* values corresponding to the first date) and using *σ*^0^ values obtained for each date. The differences (Δ*SM*) between the inverted *SM* values and the initially considered conditions (0.05, 0.20 and 0.35 cm^3^cm^-3^) for the different dates were computed. Figure 16 shows the Δ*SM* values obtained in both C- and L-bands considering 30° incidence angle, VV polarization and a soil moisture of 0.20 cm^3^cm^-3^. On the first dates variations were quite erratic as in the case of Δ*σ*^0^. Nevertheless, on the last date all the fields showed lower *SM* values than initially assumed (Δ*SM* values below zero), especially in L-band. The same analysis was carried out in the different sensor configurations and moisture conditions assumed. Tables 5 and 6 summarize the variations (average, minimum and maximum) in the inverted *SM* between the first and last date on the different conditions, for both C- and L-bands.

The results obtained suggest that roughness temporal dynamics could cause the inverted *SM* to be underestimated (negative *ASM̅* values) if constant (initial) roughness parameters are considered along the growing season. The underestimations seem more severe if the incidence angle is large and the soil is wet. In fact at low incidence angles, especially in C-band, variations could be positive, causing soil moisture to be overestimated. This effect may be a result of the smoothening of soils that caused the *σ*^0^ (and hence the inverted *SM*) to increase at steep incidence angles and decrease at large angles as a consequence of a more specular-like behaviour of the surface.

Variations were in the same range in both C- and L-bands, but they were slightly larger in L-band. Underestimations could reach values higher than 0.10 cm^3^cm^-3^, which is an error value that cannot be ignored from an applications' point of view.

## 4. Conclusions

The results of this synthetic study suggest that both field scale roughness spatial variability and precipitation induced temporal variations are aspects that need to be taken into account when inverting backscatter to soil moisture.

In addition, both effects seem to be strongly influenced by the incidence angle and frequency of the observations. The accuracy of the *SM* retrievals varied also strongly depending on the moisture content of soils, with highest errors observed over wet conditions.

Regarding roughness spatial variability a standard deviation of *s* and *l* of respectively 0.30 cm and 19.0 cm was observed. This variability could cause an error in the calculated *σ*^0^ of approximately 1-3 dB, depending on the acquisition parameters. Such an error would cause an *rmse* in the retrieval of *SM* between 0.05 and 0.20 cm^3^cm^-3^, approximately. Lowest errors were observed at intermediate incidence angles of around 25°-30° for C-band and 15°-20° for L-band and dry soil conditions.

Concerning roughness temporal variations, even if the reductions of *s* and increments of *l* seem minor, both effects appeared to contribute to a more specular like behaviour of the soil surface, that led to an increase in the *σ*^0^ at low incidence angles and a decrease at large angles. As a consequence, significant underestimations of *SM* (even higher than 0.10 cm^3^cm^-3^) could be expected if *s* and *l* are considered constant and incidence angles are medium or large. The opposite seems to occur at steep angles where the *SM* could be overestimated. These results suggest that assuming constant roughness conditions along a growing cycle could cause severe inaccuracies in the retrieved *SM* values.

In summary, the analysis indicates that both the spatial variability and temporal dynamics of surface roughness could cause severe inaccuracies in the retrieval of soil moisture from SAR observations. So far, a very precise characterisation of roughness needs to be carried out in order to obtain sufficiently accurate moisture estimations from SAR data. The results of this analysis should be completed with experimental observations in future studies.

## Figures and Tables

**Table 1.** Tillage class of the test fields on the different measurement dates.

**Test field**	**22/09/2004**	**08/10/2004**	**24/10/2004**	**12/11/2004**	**28/11/2004**	**17/12/2004**	**01/03/2005**
188	Rough Harrowed	Rough Harrowed	Roller Compacted	Seedbed	Seedbed	Seedbed	Seedbed
189	Rough Harrowed	Rough Harrowed	Smooth Harrowed	Seedbed	Seedbed	Seedbed	Seedbed
193	Rough Harrowed	Rough Harrowed	Seedbed	Seedbed	Seedbed	Seedbed	Seedbed
194	Harvested Crop	Rough Harrowed	Rough Harrowed	Seedbed	Seedbed	Seedbed	Seedbed
199	Mouldboard Plough	Mouldboard d Plough	Mouldboard d Plough	Seedbed	Seedbed	Seedbed	Seedbed
201	Smooth Harrowed	Smooth Harrowed	Roller Compacted	Seedbed	Seedbed	Seedbed	Seedbed
208	Mouldboard Plough	Mouldboard d Plough / Rough Harrowed	Mouldboard d Plough / Rough Harrowed	Roller Seedbed	Roller Seedbed	Roller Seedbed	Roller Seedbed
235	Smooth Harrowed	Smooth Harrowed	Seedbed	Seedbed	Seedbed	Seedbed	Seedbed
255	Smooth Harrowed	Smooth Harrowed	No Data	Seedbed	Seedbed	Seedbed	No Data
258	Rough Harrowed	No Data	No Data	Seedbed	Seedbed	Seedbed	No Data

**Table 2.** Field scale average roughness parameters. For each tillage class the number of fields (*N*) is given and for each parameter the average of field average values, the average of field standard deviations (*σ*) and the average of field coefficients of variation (*CV*) are shown.

		***s***			***l***		
		
**Tillage class**	***N***	*s̄*(**cm**)	*σs ¯***(cm)**	*CVs̅* (**%)**	*l̄***cm**)	*σl ¯* (**cm**)	*CVl̅* (**%**)
Harvested crop	1	1.62	0.41	25.4	20.57	11.56	56.2
Mouldboard plough	4	4.33	1.11	25.0	11.59	4.87	37.9
Harrowed rough	9	2.90	0.52	18.0	13.13	12.52	73.2
Harrowed smooth	7	2.33	0.39	16.5	17.59	14.71	77.7
Rolled	2	1.63	0.26	15.9	23.67	20.62	94.5
Seedbed	36	1.79	0.33	18.4	28.62	19.24	75.3
Rolled seedbed	4	1.47	0.27	18.3	31.10	11.16	41.9

**Table 3.** Field scale average root mean square error (*rmse*) of the autocorrelation function (*ACF*). For each tillage class the average *rmse* of the *ACF* is given, as well as the average *rmse* of the fit to the exponential and Gaussian functions.

		***rmse***	
		
**Tillage class**	***rmse****ACF̅*	**Exponential**	**Gaussian**
Harvested crop	0.087	0.113	0.217
Mouldboard plough	0.113	0.043	0.092
Harrowed rough	0.128	0.096	0.153
Harrowed smooth	0.124	0.127	0.193
Rolled	0.104	0.157	0.236
Seedbed	0.130	0.122	0.211
Rolled seedbed	0.085	0.095	0.177

**Table 4.** Average Δ*σ*^0^, minimum (Δ*σ*^0^_min_) and maximum (Δ*σ*^0^_max_) backscatter variations due to temporal roughness dynamics along the research period for the different configurations considered.

	**Incidence angle and polarization**					

	15°		30°		45°	

	VV	HH	VV	HH	VV	HH
**C-band**$\bar{{\Delta \sigma }^{0}}$	**1.09**	**1.08**	**-1.21**	**-1.36**	**-2.18**	**-2.67**
Δ*σ*^0^_min_	-0.78	-0.97	-2.79	-2.82	-6.93	-7.62
Δ*σ*^0^_max_	5.55	5.52	-0.11	0.06	-0.31	0.16

**L-band** $\bar{{\Delta \sigma }^{0}}$	**-1.36**	**-1.31**	**-2.41**	**-2.34**	**-2.77**	**-2.77**
Δ*σ*^0^_min_	-2.43	-2.41	-6.82	-6.32	-8.50	-7.95
Δ*σ*^0^_max_	0.16	0.22	-0.05	0.13	-0.11	0.20

**Table 5.** Average Δ*SM̅*, minimum (Δ*SM*_min_) and maximum (Δ*SM*_max_) soil moisture variations for the different configurations considered in C-band.

	**Incidence angle and polarization**					

**C-band**	15°		30°		45°	

SM	VV	HH	VV	HH	VV	HH
0.05 cm^3^cm^-3^ Δ*SM̅*	**0.05**	**0.05**	**-0.04**	**-0.04**	**-0.04**	**-0.04**
Δ*SM*_min_	-0.05	-0.05	-0.05	-0.05	-0.05	-0.05
Δ*SM_max_*	0.27	0.30	-0.01	-0.01	-0.01	0.01

0.20 cm^3^cm^-3^ Δ*SM̅*	**-0.06**	**-0.07**	**-0.05**	**-0.08**	**-0.10**	**-0.11**
Δ*SM_min_*	-0.20	-0.20	-0.11	-0.20	-0.20	-0.20
Δ*SM_max_*	0.00	0.01	-0.01	0.00	-0.01	0.02

0.35 cm^3^cm^-3^ Δ*SM̅*	**-0.11**	**-0.12**	**-0.10**	**-0.12**	**-0.12**	**-0.20**
Δ*SM_min_*	-0.35	-0.35	-0.19	-0.23	-0.29	-0.35
Δ*SM_max_*	0.01	0.01	-0.01	0.01	-0.03	0.03

**Table 6.** Average Δ*SM̅*, minimum (Δ*SM*_min_) and maximum (Δ*SM*_max_) soil moisture variations for the different configurations considered in L-band.

	**Incidence angle and polarization**					

**L-band**	15°		30°		45°	

SM	VV	HH	VV	HH	VV	HH
0.05 cm^3^cm^-3^ Δ*SM̅*	**-0.03**	**-0.04**	**-0.04**	**-0.04**	**-0.04**	**-0.04**
Δ*SM_min_*	-0.05	-0.05	-0.05	-0.05	-0.05	-0.05
Δ*SM_max_*	0.00	0.00	0.00	-0.01	0.00	0.01

0.20 cm^3^cm^-3^ Δ*SM̅*	**-0.06**	**-0.06**	**-0.09**	**-0.09**	**-0.12**	**-0.12**
Δ*SM_min_*	-0.10	-0.11	-0.20	-0.19	-0.20	-0.20
Δ*SM_max_*	0.01	0.02	0.00	0.01	-0.01	0.02

0.35 cm^3^cm^-3^ Δ*SM̅*	**-0.11**	**-0.11**	**-0.15**	**-0.19**	**-0.17**	**-0.15**
Δ*SM_min_*	-0.19	-0.20	-0.35	-0.35	-0.35	-0.35
Δ*SM_max_*	0.02	0.03	-0.01	0.02	-0.01	0.04
